# Depressive symptoms across the retirement transition in men and women: associations with emotion regulation, adjustment difficulties and work centrality

**DOI:** 10.1186/s12877-024-05228-2

**Published:** 2024-07-31

**Authors:** Sara Hed, Anne Ingeborg Berg, Isabelle Hansson, Marie Kivi, Margda Waern

**Affiliations:** 1 Institute of Neuroscience, Department of Psychiatry, Sahlgrenska Academy, University of Gothenburg, SU/Sahlgrenska, Blå Stråket 15, 41345 Gothenburg, Sweden; 2 Dept of Neuropsychiatry, Region Västra Götaland, Sahlgrenska University Hospital, Gothenburg, Sweden; 3 Centre for Ageing and Health, University of Gothenburg, Gothenburg, Sweden; 4 Department of Psychology, University of Gothenburg, Gothenburg, Sweden; 5 Psychosis Department, Region Västra Götaland, Sahlgrenska University Hospital, Gothenburg, Sweden

**Keywords:** Depressive symptoms, Gender, Longitudinal, Population-based, Retirement transition, Emotion regulation

## Abstract

**Background:**

Retirement is a major life event and factors driving depression in the retirement transition might differ in men and women. The aim was to prospectively study depressive symptoms across the retirement transition in men and women and to test associations with emotion regulation strategies (suppression and reappraisal), adjustment difficulties, and work centrality.

**Methods:**

The sample included 527 individuals from the population-based Health, Aging and Retirement Transitions in Sweden (HEARTS) study who were working at baseline and retired during one of the following four annual measurement waves. Participants contributed with a total of 2635 observations across five measurement waves. Depressive symptoms were measured with the Center for Epidemiologic Studies Depression Scale (CES-D); total score was modelled as a function of time to and from retirement. Changes over the retirement transition were analyzed with multilevel growth curve models. Gender differences in associations with emotion regulation, adjustment difficulties and work centrality were examined by including interaction effects with sex.

**Results:**

We observed a general reduction of depressive symptoms in the early years of retirement in both men and women. Higher suppression was related to higher depression scores while higher cognitive reappraisal was related to lower levels of depressive symptoms. Women more often used cognitive reappraisal, and men more often suppression, but no significant gender interaction in associations with depressive symptoms could be shown. Retirement adjustment difficulties and greater importance of work for self-esteem were related to higher depression scores. Greater meaning of work, on the other hand, was related to lower levels of depressive symptoms, and this association was stronger in men.

**Conclusions:**

There was a general reduction of depression scores in the early years of retirement in both women and men. Findings suggest that basing one’s self-esteem on workplace performance was related to higher levels of depressive symptoms after retirement, while perceiving one’s job as important and meaningful may facilitate better adjustment in terms of lower depression symptom levels, especially in men.

**Supplementary Information:**

The online version contains supplementary material available at 10.1186/s12877-024-05228-2.

The retirement transition is a major life event with substantial changes in everyday life. Understanding how people cope with these changes, including the factors involved in shaping mental health trajectories after retirement, is important to meet the needs of rapidly aging populations. The retirement transition may be more challenging for some persons than others [1, 2]. While some studies [3–5] suggest that retirement may impact negatively on mental health, others show limited [6, 7] or even beneficial [8–11] effects. Declining rates of antidepressant use after retirement have also been reported [12]. The authors of a recent meta-analysis demonstrated a nearly 20% reduction in the risk of depressive symptoms or depression in connection with retirement. They suggested that in order to inform and adapt mental health-promoting strategies in both clinical and public health contexts, the contributions of individual and environmental characteristics need to be teased out [8].

## Drivers of depression in the retirement transition

Retirement is a highly heterogenous process [13] and previous studies suggest that difficulties adjusting to retirement could potentially cause an increase in depressive symptoms [14] and reduced retirement satisfaction [15]. To measure difficulties in adjusting to the loss of the work role, previous studies have investigated negative experiences in the retirement transition including missing social contacts and changes in work-life status [16]. Adjustment has been the focus of many studies [17], it is possible for individuals to adjust without experiencing well-being in life as a retiree [15]. The resource-based dynamic model postulates that an individual’s access to resources defines both the conditions and quality of the retirement experience [18]. Recognizing the dynamic nature of the retirement transition, Wang et al. (2011) argue that changes in environmental and psychological resources are the underlying mechanisms which cause fluctuation of well-being in the retirement process [18]. Difficulties adapting to the loss of the work role and work-related social ties decrease well-being in retirement, and the process of getting used to altered life circumstances in retirement is referred to as retirement adjustment [15].

Work centrality may also play a role in shaping psychological experiences during retirement. This concept refers to the importance of work in an individual’s life [19]; high work centrality means that work and the work role is an important aspect of life and also an important aspect of the individual’s identity [20]. Attachment to the work role has been identified as an important pre-retirement resource and the loss of the work role has been found to exacerbate depressive symptoms [14] and poor quality of life in retirement, especially in the absence of alternative roles [21].

Cognitive emotion regulation strategies (e.g., suppression, reappraisal) may also be important resources for mental health during the retirement transition as such strategies are involved in the onset and maintenance of depression [22]. Emotional coping is central to balance negative and positive emotions [23]. Maladaptive emotion regulation strategies (e.g., rumination, suppression) are associated with overall psychopathology; adaptive strategies (e.g., acceptance, reappraisal) are related to lower levels of psychopathology [24, 25]. Hu et al. (2014) examined the relationship between emotion regulation and mental health in a meta-analysis and found that reappraisal was positively correlated with mental health while suppression was negatively correlated [26]. Hence, individuals who habitually used suppression as a regulation strategy were more prone to depressive symptoms compared to those who more frequently used reappraisal. The latter would promote psychological well-being [25]. Several studies have examined depression and well-being in connection with retirement [8, 14, 17, 27] but we could identify no studies providing data on emotion regulation strategies and depression during the retirement transition. How we cope with and adjust to stressful events is dependent on our cognitive appraisal- our subjective interpretation of the stimuli [28] and we need to know more about how people manage emotions in this transitional phase.

## Gender-specific predictors of depression in the retirement transition

While depression is more common in young women than men [29], some studies suggest that the gender gap decreases with age [30]. Gender-stratified analyses within the systematic review and meta-analysis of Odone and colleagues [8] showed no significant gender difference in the prevalence of depressive symptoms in connection with retirement. Nevertheless, it is possible that men and women may have differential experiences of the retirement transition.

While women more often enter retirement with higher levels of depressive symptoms [14], one study indicated that being fully retired was associated with higher levels of psychological distress in men (age 65–74) [31]. In a traditional work-force gender pattern, women are more likely to experience loss of roles and status at various points throughout adult life, but the role expectations on men are more consistent [32]. This might in part explain differential experiences of retirement in men and women. In a recent cross-sectional study [33] we observed similar depression scores in newly retired men and women, but results suggested that several factors (health, quality of social network, social support and competence satisfaction) were associated with depression in men but not in women.

Longitudinal studies are needed to further disentangle potential gender-specific patterns in the development of depressive symptoms across the retirement transition. Several recent longitudinal studies investigated depressive symptoms in relation to the retirement transition in Sweden [7, 11, 34–36] but to our knowledge only Nyberg et al. presented gender-specific analyses [7]. Similar sociodemographic risk factors were associated with high-scoring depression groups in men and women, but men who were unmarried were more likely to be found among the groups with higher depression scores [7], suggesting that marriage was protective in men but not in women.

Gender-specific predictors of depression in the retirement transition are likely tied to the circumstances and conditions of the labour market and pension system. Rydberg et al. (2019) analysed data from face-to-face interviews with a population-based sample of 70-year-olds, carried out in Sweden during 2014–2016, and reported that the prevalence of any depression (either major or minor) was 8.9% (6.6% for men and 10.9% for women) [37]. That study could not elucidate whether the observed gender difference might in part be related to retirement transition issues. Sweden is a strong welfare state with a flexible pension system [38]. For the cohort included in this study, the national pension scheme allowed public pension withdrawal from age 61 and employees had the right to remain employed until age 67; these age limits have now been increased to 63 and 69, respectively [39]. There are some indications that people with limitations in physical functioning, especially women, retire at a higher age nowadays [40], and this might be related to institutional factors including strong financial incentives to continue working. This issue is also raised by König (2017) who points out that the negative effects of a more flexible and privatized pension system affect women to a greater degree than men [41].

## The current study

As the driving factors of depression in the retirement transition might differ in men and women [33], a longitudinal approach to the investigation of factors of potential importance might contribute to a better understanding of depressive symptoms in connection with retirement. The aim of our study was to investigate level of and change in depressive symptoms across the retirement transition, and potential associations with emotion regulation strategies, adjustment difficulties, and work centrality, using data from the longitudinal population-based HEalth, Aging and Retirement Transitions in Sweden (HEARTS) study [35]. More specifically, we examined if individuals who experienced greater retirement adjustment difficulties and stronger pre-retirement work centrality showed higher levels of depressive symptoms following retirement. Work centrality encompasses not only the importance of work performance for the individual’s self-esteem [42], but also how meaningful, important and motivating work is perceived [43]. Building on the knowledge that maladaptive emotion regulation strategies are components associated with depression [24, 25], we hypothesized that suppression as an emotion regulation strategy is related to higher levels of depressive symptoms following the retirement transition and that reappraisal as an emotion regulation strategy is related to lower levels of depressive symptoms. In order to identify possible gender-specific patterns, we employed an explorative approach and assessed interaction effects between sex and each of the predictor variables. Given the dearth of gender-specific data on predictors of depression in the retirement transition, we also conducted supplementary stratified analyses. Due to a lack of previous research in the area, we made no assumptions regarding specific gendered patterns in the predictor variables.

## Materials and methods

### Sample and procedure

The HEARTS study is a longitudinal population-based study including older adults drawn from the Swedish population registry. A total of 5913 individuals aged 60–66 participated at the first data collection in 2015, resulting in a response rate of 39%. The study is survey-based with annual follow-ups and the retention rate for the years 2016–2019 was 67–79%. The survey was conducted online, but a paper version was also offered. In the first wave, 68.8% chose to respond online and 31.2% used the paper version. Questionnaires include measures of retirement status, perceptions of work life, health, lifestyle, well-being, cognitive function, social network and personality. The rationale for the study, research protocol, and initial findings are described in Lindwall et al. (2017) [35], and more information about the study design, measurement domains, and previous publications can be found on the Open Science Framework (OSF) project page (https://osf.io/wcbxu/). The study was approved by the ethical approval board at the University of Gothenburg, diary no. 970–14. The sample is generally representative of the population in Sweden born 1949–1955, however a greater proportion of women responded to the survey and the HEARTS cohort consist of a larger proportion of individuals with tertiary education compared to the general population (sample = 41%; population 33%) [6, 35].

In the present study we used a data sample drawn from the first five waves (2015–2019) of the HEARTS study. In order to assess changes in depressive symptoms relative to the retirement transition, we included participants who were working at baseline (*n* = 3841) and retired during the study period. Retirement status was measured with the following question ‘Are you retired (receive old-age pension)?’ with the following response alternatives: (1) no, (2) yes, but still working and consider myself a worker, (3) yes, still working but consider myself a retiree, and (4) yes, full-time retiree. Participants were included if they were not yet retired (1) at baseline (*n* = 3139) and retired fully (transitioned from 1 to 4) during one of the following measurement waves (2016–2019; *n* = 1253). Participants were excluded if they retired gradually (e.g., transitioned from 1 to 2 or 3 to 4, *n* = 322) or reversed their transition (e.g. from 2/3/4 to 1 or 4 to 3, *n* = 134) at some point during the study period. Participants were also excluded if they reported not working due to unemployment or disability the year before they retired (*n* = 52), or if they did not complete all five measurement waves (in which case we would not be able to determine their retirement status over the entire period, *n* = 218). The final sample consisted of 527 individuals who contributed with a total of 2635 observations. Retirement occurred between W1 and W2 in 156 individuals. Corresponding figures were 149 between W2 and W3, 126 between W3 and W4 and 96 between W4 and W5.

The sample drawn for the present study did not differ significantly from those excluded due to gradual, reversed, indirect, or incomplete transition pathways in terms of gender, education, relationship status, spouse’s work status, health, or depressive symptoms (see Supplementary Material, Table S1). The sample was however marginally younger (mean age 62.7) than those excluded (mean age 62.9) (*p* = 0.008).

### Measures

#### Depressive symptoms

Depressive symptoms were rated at all five measurement occasions using an 11-item short form [44] of the Center for Epidemiological Studies-Depression Scale (CES-D) [45]. This version has previously been employed in a Swedish population-based study of older adults. For each item, respondents rated how often during the past week they had experienced each symptom (e.g. “I was bothered by things that usually don’t bother me”). Response alternatives were grouped by number of days (“0 days/less than 1 day” (0), “1–2 days” (1), “3–4 days” (2), and “5–7 days” (3)), and a sum score was calculated (range 0–30). Cronbach’s alpha ranged between 0.72 and 0.78 over the 5 study waves (W1: 0.76; W2: 0.77; W3: 0.72; W4 0.75; W5: 0.78).

#### Emotion regulation strategies – suppression and reappraisal

To assess emotion regulation strategies, we used the 10 Item Emotion Regulation Questionnaire (ERQ) [25]. This scale was included in wave 5 and consists of two subdomains; reappraisal (6 items; e.g. “I control my emotions by changing the way I think about the situation I’m in”) and suppression (4 items; e.g. “I control my emotions by not expressing them”). The items were rated on a scale from strongly disagree (1) to strongly agree (7) and a mean score was calculated for each participant. Cronbach’s alpha for the reappraisal scale was 0.84 and for the suppression scale 0.69.

#### Adjustment difficulties

To measure adjustment difficulties, we selected items from the subscale of negative experiences in retirement from the Retirement Experience Questionnaire (REQ) [46]. The included items were “I don’t have many meaningful tasks or roles now that I am retired”, “I miss the socialising and day-to-day structure of working life”, “I am finding it hard to adjust to being retired.” An additional item was added by the developers of the HEARTS study, “I miss my work identity”. We excluded the two negative items aimed to assess anxiety and financial limitations. The rational for doing so was that anxiety may correlate with depression and financial limitations may capture an aspect of adjustment difficulties other than that we aimed to assess. The items were rated on a scale from 1 (completely false) to 5 (completely true) and a mean score was calculated. This measure was included in all waves and the alpha reliability values were W2, 0.75; W3, 0.76; W4, 0.78; W5, 0.73.

#### Work centrality

Two facets of work centrality were assessed: importance of work for one’s self-esteem and meaning of work. Four items from the Importance of Performance to Self-Esteem scale (IPSE) [42] were taken from baseline (W1; i.e., before retirement): “Doing well at work gives me a sense of self-respect”, “My self-esteem is influenced by my workplace performance”, “I feel better about myself when I know I am doing well at work”, and “I feel bad about myself whenever my work performance is lacking”. The items were rated on a 7-point scale from strongly disagree (1) to strongly agree (7) and a mean score was calculated. The reliability Cronbach’s alpha was 0.78. To measure the meaning of work we used W1 data from a three-item subscale of from the Copenhagen Psychosocial Questionnaire aimed to assess meaning of work (COPSOQ) [43]. These were “My work is meaningful”, “I feel that the work I do is important” and “I feel motivated and involved in my work”. The items were rated on a 5-point scale ranging from completely false (1) to completely true (5) and a mean score was calculated. The reliability Cronbach’s alpha was 0.86.

### Covariates

We controlled for potential confounding effects of sociodemographic variables: age at baseline, education (in years), relationship status (partner = 1, no partner = 0), and partner’s retirement status (working = 1, not working = 0). To evaluate the robustness of our findings, we further included measures on self-rated functional health and social support as these are well-established factors with considerable impact on well-being and retirement adjustment. Social support was measured with the Multidimensional Scale of Perceived Social Support (MSPSS) [47]. The scale consists of 12 items and three subdomains, each with four items: social support from friends, family and significant others (e.g., “There is a special person who is around when I am in need”). Respondents rated the items on a 7-point scale ranging from completely false (1) to completely true (7) and a mean score was calculated. Self-rated functional health was measured with a single item to capture perceived functional limitations due to health problems (”Does your general health condition prevent you from doing the things you want to do?”). The response alternatives were “Not at all” (1), “Yes, to some extent” (2) or “Yes, to a great extent” (3). Age and education were measured at baseline while relationship status, partner’s retirement status, social support (α = 0.95–0.96) and self-rated functional health were assessed in all five waves.

### Statistical analyses

We calculated descriptive statistics and used an independent sample t-test or chi-squared test to test for gender differences in all variables. Changes in depressive symptoms over the retirement transition were analyzed with multilevel growth curve models using the lmer function of the lme4 package (version 1.1–23; (Bates et al. 2015) [48] in R (version 3.6.2; R Core Team, 2019). The annual measurements were restructured as conditioned on the wave in which the participants reported their retirement, ranging from four years before (-4, -3, -2, -1) to four years after (0, 1, 2, 3) retirement. CES-D score was modelled as a function of time to and from retirement with the intercept centred on the wave corresponding to the year that followed retirement. To account for non-linear trajectories over the retirement transition, we evaluated both linear, quadratic, and cubic slopes.

Associations between depressive symptoms and the predictor variables were examined in a stepwise fashion. The first model included age, education, relationship status, and spouse’s retirement status. Suppression and reappraisal ratings were added in the second model. In the third model, we added adjustment difficulties, importance of work for self-esteem, and meaning of work. In the fourth and final model, we added social support and self-rated functional health to test the robustness of our results and to gain an increased understanding of the relationships among the variables in our model. Gender differences in associations between depressive symptoms and emotion regulation, adjustment difficulties, and work centrality were examined by including interaction effects between sex and each of the predictor variables. In supplementary analyses we created separate models for men and women. All predictors were modelled as fixed effects and continuous variables were cantered on the mean. The variables age, education, importance of work for self-esteem, and meaning of work were derived from baseline (wave 1). Relationship status, spouse’s retirement status, adjustment difficulties, social support, and self-rated functional health were derived from one year after retirement. For participants who retired between the fourth and fifth wave of data collection (i.e., only providing one post-retirement assessment), we included measures from the year of retirement.

## Results

Descriptive statistics of the study variables are presented separately for men and women in Table 1. Slightly over half (56%) were women. Women had slightly more years of education (13.80) compared to men (13.21). Approximately 80% of the sample had a partner, and men were more likely to have a partner who was still working. Men more often used suppression as a regulation strategy; women more often used cognitive reappraisal. Men reported significantly more adjustment difficulties compared to women. Women reported greater meaning of work compared to men, but there was no difference between the sexes in importance of work for self-esteem. Women perceived more social support compared to men. Men and women reported similar levels of subjective functional health.

**Table 1:** Descriptive statistics of the study variables in the sample, and by gender

	**All** **M (SD) /%**	**Women** **M (SD)/%**	**Men** **M (SD)/%**	Group difference *t/* ${{\varvec{X}}}^{2}$
n	*527*	*297*	230	
Education years	13.6 (3.4)	13.8 (2.9)	13.2 (3.5)	-2.1*
Age at first wave	62.7 (1.6)	62.6 (1.6)	62.7 (1.7)	1.2
Partner (% yes)	80.2%	79.5%	80.4%	0.0
Partner working (% yes)	25.1%	14.1%	37.4%	37.6***
Suppression	3.1 (1.1)	2.9 (1.1)	3.4 (1.1)	5.2***
Reappraisal	4.5 (1.1)	4.7 (1.1)	4.3 (0.9)	-3.4***
Adjustment difficulties	1.2 (0.5)	1.1 (0.5)	1.3 (0.5)	3.4***
Importance of work for self-esteem	5.2 (1.2)	5.2 (1.2)	5.1 (1.2)	-0.8
Meaning of work	4.2 (0.8)	4.3 (0.8)	4.1 (0.8)	-2.9***
Social support	6 (1.3)	6.2 (1.2)	5.7 (1.4)	-3.9***
Self-rated functional health	1.4 (0.6)	1.4 (0.6)	1.4 (0.6)	0.2
CES-D score W1	4.7 (4.3)	4.9 (4.5)	4.4 (4)	0.2
CES-D score W2	4.3 (4.2)	4.4 (4.3)	4.2 (4.1)	0.5
CES-D score W3	3.9 (3.8)	4.0 (4)	3.7 (3.5)	0.3
CES-D score W4	3.8 (3.9)	3.8 (3.9)	3.7 (3.9)	0.9
CES-D score W5	3.7 (4.1)	3.8 (4.3)	3.7 (3.9)	0.8

^*^p < 0.05; ***p < 0.001

**Fig. 1:**
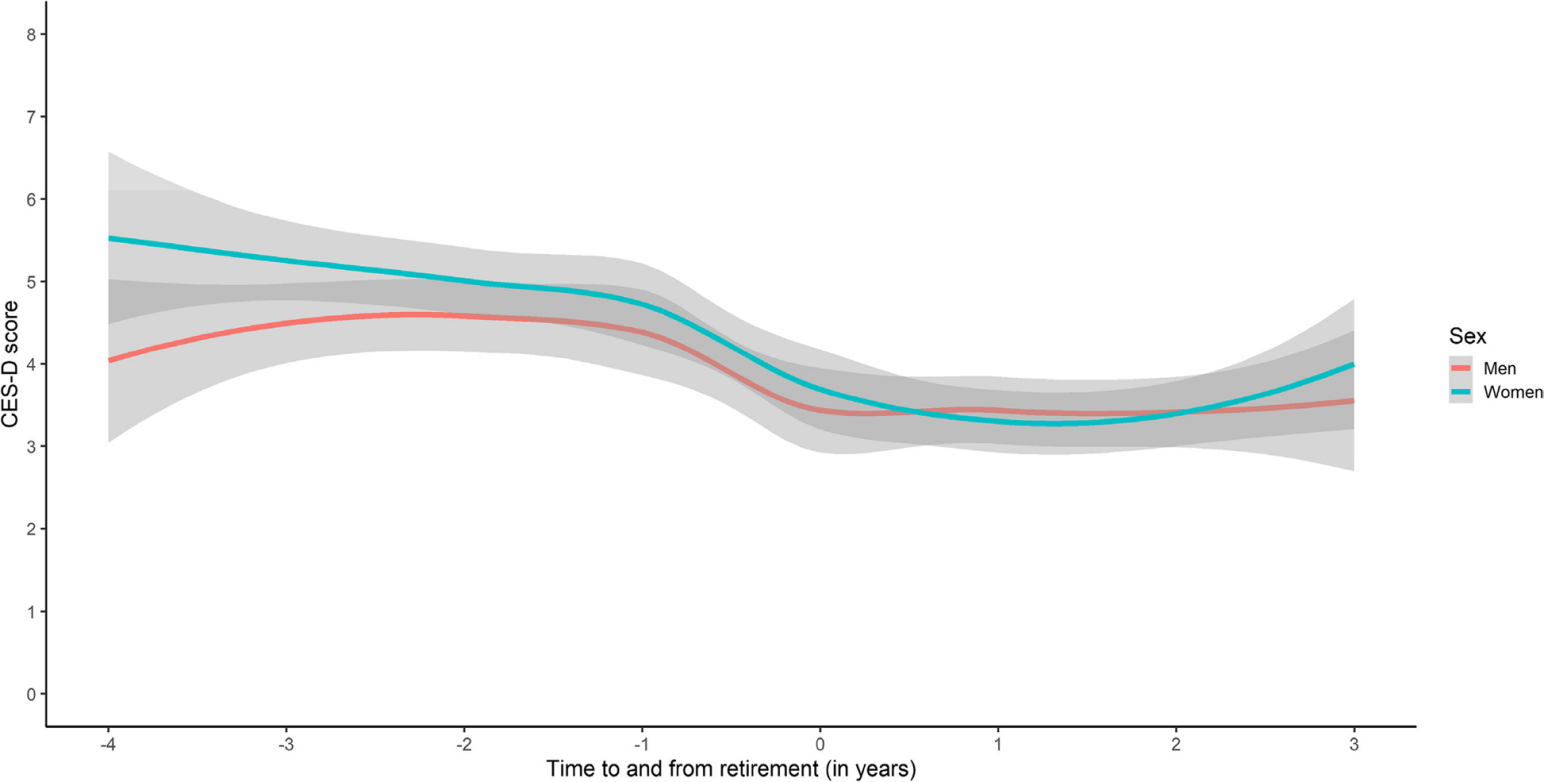
Trajectories of depression symptoms (CES-D*) in men and women** in relation to the retirement transition, a population-based sample. Note. The shaded area illustrates the confidence interval. * CES-D = Center for Epidemiologic Studies Depression Scale.**Women: 297 Men: 230

CES-D scores did not differ in men and women at any study wave. Analysis of changes over time showed a general reduction of depressive symptoms over the retirement transition in both men and women (Fig. 1). Women had slightly higher depression scores before retirement, and a slightly steeper decrease after retirement, but these differences were not significant (Table 2). The growth curve model showed a significant cubic trend for both men and women (see Table 3 for general effect and Supplementary Table S3 for gender-stratified analyses), with stability before retirement, a decrease over the transition period, and stability after retirement (as illustrated in Fig. 1).

**Table 2:** CES-D scores in relation to the retirement event

	Time to and from retirement							
	-4	-3	-2	-1	0	1	2	3
Men								
* n*	46	93	154	218	222	170	125	64
* M*	3.98	4.62	4.42	4.39	3.44	3.76	3.20	3.66
* SD*	3.98	4.31	4.02	3.78	3.77	3.94	3.75	3.86
Women								
* n*	45	114	203	273	285	234	167	86
* M*	5.91	4.90	4.97	4.73	3.69	3.34	3.37	4.01
* SD*	5.58	4.35	4.45	4.34	4.10	3.57	3.59	4.59
*Group difference t*^a^	-1.9	-0.46	-1.2	-0.91	-0.71	1.08	-0.39	-0.50

^a^There were no differences between men and women in accordance with the t-test

**Table 3:** Estimates of associations between depressive symptoms and emotion regulation, adjustment difficulties, and work centrality

	CES-D score			
	*b* (*SE*)	*b* (*SE*)	*b* (*SE*)	*b* (*SE*)
Time	-0.28*** (0.06)	-0.29*** (0.06)	-0.27*** (0.06)	-0.25*** (0.06)
Time^2^	0.16*** (0.04)	0.16*** (0.04)	0.15*** (0.04)	0.15*** (0.04)
Time^3^	0.04*** (0.01)	0.04*** (0.01)	0.04*** (0.01)	0.03*** (0.01)
Gender (male = 0, female = 1)	0.46 (0.32)	0.79*(0.34)	1.16***(0.31)	1.27***(0.27)
Education	0.03 (0.05)	0.08 (0.05)	0.06 (0.05)	0.02 (0.04)
Age	0.07 (0.09)	0.03 (0.10)	-0.03 (0.09)	-0.03 (0.08)
Partner	-1.80*** (0.41)	-1.62*** (0.42)	-1.48*** (0.40)	-1.48*** (0.40)
Partner working	0.45 (0.37)	0.53 (0.39)	0.52 (0.36)	0.41 (0.30)
Supression		0.41** (0.15)	0.25 (0.14)	0.03 (0.12)
Reappraisal		-0.46** (0.15)	-0.32* (0.15)	-0.12 (0.13)
Adjustment difficulties			2.12*** (0.28)	1.31*** (0.25)
Importance of work for self-esteem			0.50*** (0.13)	0.36*** (0.11)
Meaning of work			-0.99*** (0.19)	-0.73*** (0.16)
Social support				-0.95*** (0.11)
Self-rated functional Health				1.53*** (0.22)
Constant	4.56*** (0.41)	4.18*** (0.42)	3.84*** (0.40)	3.19*** (0.36)
Observations	2311	2183	2073	2073
Log likelihood	-5,983.67	5,659.25	-5,319.83	-5,001.77
Akaike information criterion	11,989.34	11,344.51	10,671.66	10,039.55
Bayesian information criteron	12,052.54	11,418.46	10,761.85	10,140.20

^*^p < 0.05; **p < 0.01; ***p < 0.001

Having higher suppression scores was related to higher CES-D scores while higher cognitive reappraisal was related to lower levels of CES-D scores (Table 3). Analyses of gender differences indicated a stronger association between suppression and depressive symptoms in men, but this difference was not significant (*b*_interaction_ = -0.17, *SE* = 0.29, *p* > 0.05, See Supplementary Material, Table S2). No significant gender difference was found in the association between cognitive reappraisal and depressive symptoms (*b*_interaction_ = 0.004, *SE* = 0.32, *p* > 0.05). The inclusion of adjustment difficulties, importance of work for self-esteem, and meaning of work reduced the effect sizes, resulting in a non-significant effect of suppression. When controlling for social support and self-rated functional health, none of the two emotion regulation strategies were significantly associated with depressive symptoms.

Having retirement adjustment difficulties was related to higher levels of depressive symptoms. This association was stronger in women, but the difference in effect size was not significant (*b*_interaction_ = 1.03, *SE* = 0.55, *p* > 0.05). Greater importance of work for self-esteem was also related to higher levels of depressive symptoms, and again, there was a slightly stronger effect in women but no significant gender difference (*b*_interaction_ = 0.24, *SE* = 0.25, *p* > 0.05). Greater meaning of work, on the other hand, was related to lower levels of depressive symptoms, and this association was stronger in men (*b*_interaction_ = 0.72, *SE* = 0.36, *p* < 0.05). The associations remained significant also when self-rated functional health and social support were included in the model. Greater social support and fewer health-related functional limitations were related to lower levels of depressive symptoms. Age, years of education, and partner’s retirement status were unrelated to depressive symptoms, but being in a relationship was associated with lower depression score.

While no gender differences in depressive symptoms were observed in the model without predictors, women reported higher CES-D scores than men when accounting for emotion regulation, adjustment difficulties, and work centrality, highlighting the influence of mean level differences in the predictor variables between men and women. Separate analyses for men and women (Supplementary Material, Table S3) elucidated the relative importance of meaning of work in men and women (*b*_men_ = -1.34, *SE* = 0.28, *p* < 0.001, *b*_women_ = -0.70, *SE* = 0.25, *p* < 0.01).

## Discussion

We used a longitudinal approach to shed light on gender-specific trajectories of depressive symptoms across the retirement transition and found similar levels and change in depressive symptoms in men and women, as well as a general reduction of depressive symptoms in both genders. While the symptom levels remained relatively stable in the last years before retirement and first years that followed, both men and women showed a decrease in depressive symptoms in the year of retirement. Regarding gender interactions with variables that might help to explain depressive symptoms, only meaning of work showed a significant difference between men and women.

Results expand on those of our previous cross-sectional study [33] that showed similar depression scores for men and women in the early years after retirement. Further, our results expand on previous longitudinal research [8], showing that retirement seems to be associated with a reduced risk of depressive symptoms, by demonstrating the robustness of these findings in gender-specific analyses. Our findings contrast with those of the previous gender-stratified Swedish study [7] which showed that levels of depressive symptoms were relatively unaffected by the retirement transition. Birth cohort differences might help to explain the disparity. Our sample was born 1949–1955, a decade after the sample in the study of Nyberg and colleagues [7]. Due to societal changes following the financial crisis in the 1990s, later cohorts might have experienced a more stressful work life, which in turn could result in a relief effect in the retirement transition. The results could in part also be explained by changes in several health indicators in Sweden over time [49], with increased cognitive and physical function and decreased depressive symptoms [37]. The greatest health-related changes over time were observed in women [49]. Future research on depressive symptoms during the retirement transition should further investigate differences in contexts and cohorts. More research is needed to further investigate if the positive effect of retirement on depressive symptoms is time limited as certain studies have found an increase in symptoms in the long term (10 or more years after retirement) [50].

While men in our study reported greater difficulties in adjusting to retirement compared to women, such difficulties were associated with higher levels of depressive symptoms in both genders, even after controlling for effects of physical health and social support. Our results further showed differential effects of the two work centrality measures. Interestingly, importance of work for self-esteem and meaning of work showed opposite associations with depressive symptoms. In line with our expectations, those who based their self-esteem on their performance in the workplace had higher levels of depressive symptoms after retirement. In contrast, a strong perceived meaning of work prior to retirement was related to fewer depressive symptoms after retirement. Perceiving one’s job as important and meaningful thus appears beneficial in terms of reducing the risk for post-retirement depression. This discrepancy may be understood in the light of previous research suggesting that roles that can be continued or modified in the retirement transition are helpful to adjustment, but strong role identities or a strong identification with the work role that cannot be transferred to post-retirement life might hinder a smooth transition [51, 52]. Damman and Henkens (2017) further showed that, for many retirees, work remains an important aspect of life, which may point to both adaptive and maladaptive processes [53]. The measure of meaning of work used in the current study may entail a broader aspect of work as value but also to a certain degree reflect individual resources and personality traits of importance for adjustment in the post-retirement life context. Performance on the other hand, may be more linked to short-term rewards that are simply taken away when one retires. Hence, if performing well at work means a lot to one’s sense of self-esteem, it is not favourable. On the other hand, if the workplace has provided a sense of meaning and involvement it seems to be beneficial even in the early years of retirement. The latter was more pronounced in men compared to women.

Having higher suppression scores was related to greater CES-D scores while higher cognitive reappraisal was related to lower CES-D scores, but when controlling for social support and self-rated functional health the associations were no longer significant. Women more often used cognitive reappraisal, and men more often suppression, but there were no significant gender differences in associations with depressive symptoms. While gender differences in emotion regulation strategies have been observed previously [25], our findings suggest an absence of gender differences in associations with depressive symptoms. This may need to be studied further in larger cohorts. As real-life emotion regulation involves more complex processes, which hardly could be reduced to suppression and reappraisal, more information on context and for whom suppression might play a role for depressive symptoms would be needed to better understand the process and to what extent this may differ between men and women.

## Methodological considerations

A strength of the present study was the population-based sample providing longitudinal data with annual examinations of depressive symptoms over the retirement transition. This study is one of few longitudinal studies investigating gender specific patterns in depressive symptoms during the retirement transition, and to our knowledge the first to explore associations with emotion regulation strategies. Furthermore, the study included only participants who retired during the study period. Participants who reported not working (i.e., unemployed, on sick leave, or disability pension) before they retired were excluded, as were those with gradual (i.e., working after retirement) and reversed (i.e., returning to work after retirement) transitions. Although this significantly reduced the sample size, it allowed us to identify distinct trajectories of depressive symptoms in connection with the retirement transition (i.e., modelling time to and from event). Our focus on associations with the importance of (the preretirement) work and postretirement adjustment further motivated the decision to exclude individuals who continued to work in retirement. As Sweden has a flexible pension system, this was considered necessary to maintain a sample consisting only of those who had a clearly defined work – retirement pathway. A limitation of the study is related to the fact that the HEARTS cohort is a relatively healthy population with a slightly higher educational level compared to the general population of Swedish older adults [35]. This is common in cohort studies and may have implications for the generalizability of our findings. A second point of concern is that longitudinal studies commonly have a selective attrition pattern with more frequent dropout in participants with poor health. However, the inclusion of the paper survey has contributed to counteracting such selectivity [54]. While our focus on persons with a clear-cut transition pathway meant a selection in terms of marginally younger age (an average of 2 months), there were no other differences between participants in the current study and those excluded due to gradual, reversed, indirect, or incomplete transition pathways. Importantly, there were no differences in depressive symptoms. The culture- and country-specific features of labour force policies, norms and values must be considered when comparing these results to other contexts. Gender-specific responses to the retirement transition may evolve over time, influenced by numerous factors including cultural scripts of gender and social context. Sweden is a welfare state with high employment rates among older adults as well as a larger proportion of women in the workforce compared to other countries [38, 55]. Many retirees combine work with pension, and as flexibility increases so does the variability in the retirement process [56]. In this study, we deliberately restricted our analyses to include only working individuals who retired full-time during the study period, which means that the results do not necessarily apply to individuals with deviating retirement pathways. For those unemployed or on sick leave the long-term levels of well-being may develop more negatively, after a short relief effect with increased well-being upon retirement [27]. Future studies are needed to look at groups with non-traditional retirement pathways.

## Conclusion

The findings from this population-based study show a general reduction of depression symptom scores in the early years of retirement in both women and men. Our findings suggest that basing one’s self-esteem on workplace performance may increase the risk for depression after retirement. Perceiving one’s job as important and meaningful, on the other hand, may facilitate better adjustment in terms of fewer depressive symptoms after retirement, especially among men.

## Supplementary Information

Supplementary Material 1. Table S1. Descriptive statistics of differences in baseline (wave 1) characteristics between the HEARTS cohort and the study sample. Table S2. Interaction effects of gender differences in associations between depressive symptoms and emotion regulation, adjustment difficulties, and work centrality. Table S3. Gender-stratified associations between depressive symptoms and emotion regulation, adjustment difficulties, and work centrality.

## Data Availability

The dataset supporting the conclusions of this article is available from the corresponding author on request. Data from the HEARTS study can be made available upon request and in accordance with applicable laws. For further information about accessibility of data, contact hearts@psy.gu.se.
