# Correction: Depressive symptoms across the retirement transition in men and women: associations with emotion regulation, adjustment difficulties and work centrality

**DOI:** 10.1186/s12877-024-05347-w

**Published:** 2024-09-25

**Authors:** Sara Hed, Anne Ingeborg Berg, Isabelle Hansson, Marie Kivi, Margda Waern

**Affiliations:** 1 Institute of Neuroscience, Department of Psychiatry, University of Gothenburg, SU/Sahlgrenska, Blå Stråket 15, Gothenburg, 41345 Sweden; 2 Department of Neuropsychiatry, Region Västra Götaland, Sahlgrenska University Hospital, Gothenburg, Sweden; 3 Centre for Ageing and Health, University of Gothenburg, Gothenburg, Sweden; 4 Department of Psychology, University of Gothenburg, Gothenburg, Sweden; 5 Psychosis Department, Region Västra Götaland, Sahlgrenska University Hospital, Gothenburg, Sweden

**Correction: BMC Geriatr 24**,** 643 (2024)**

10.1186/s12877-024-05228-2

Following publication of the original article [1], the authors reported that the note under Fig. 1 image should be taken out as the information is now shown in the figure caption.

Incorrect Figure:

**Figure:**
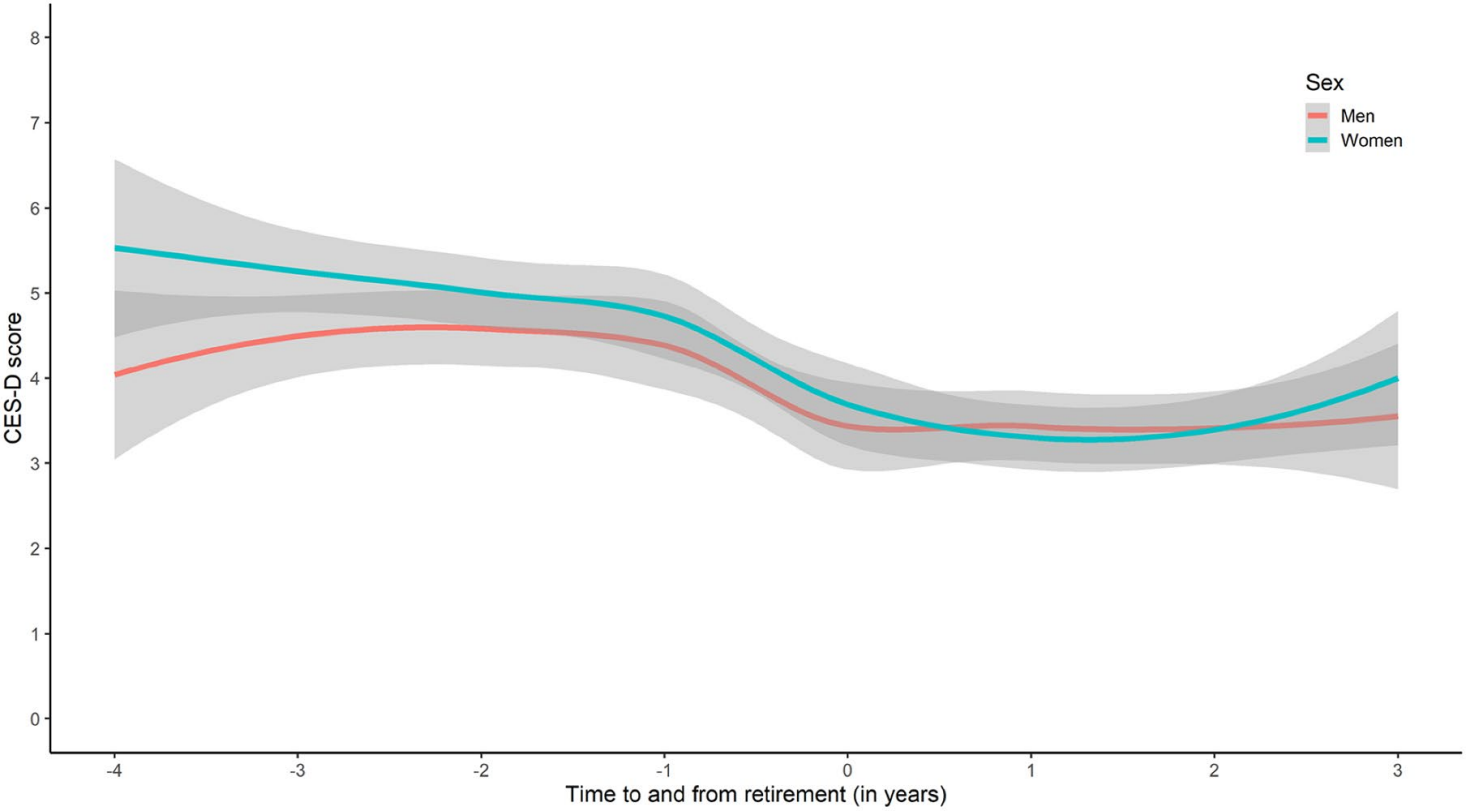
*Note*. The shade illustrates the confidence interval.* CES-D = Center for Epidemiologic Studies Depression Scale.**Women: 297 Men 230

Correct Figure:

**Fig. 1:**
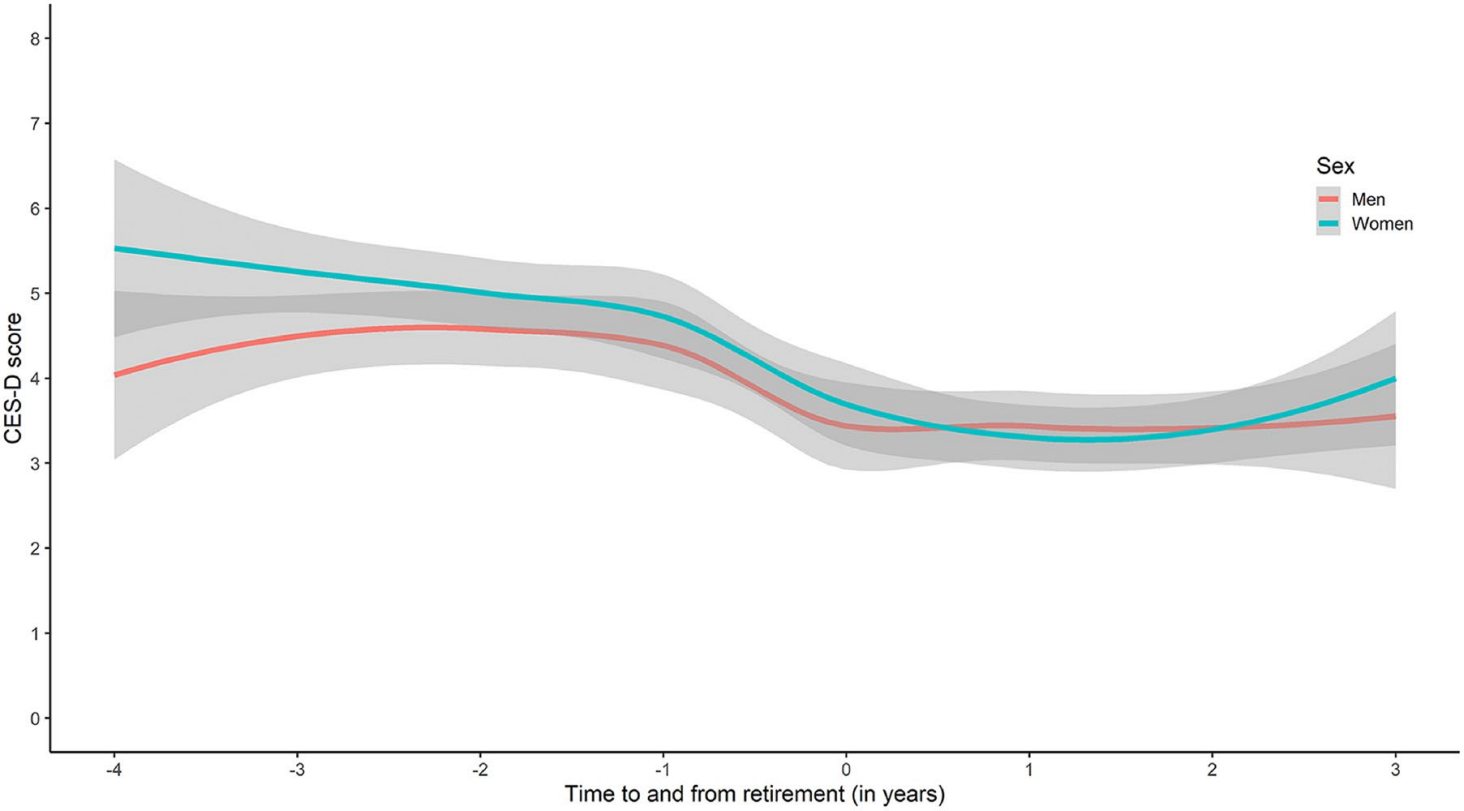
Trajectories of depression symptoms (CES-D*) in men and women** in relation to the retirement transition, a population-based sample. Note. The shaded area illustrates the confidence interval. * CES-D = Center for Epidemiologic Studies Depression Scale.**Women: 297 Men: 230

The original article [1] has been corrected.
