# Supplementary material for: Depressive symptoms across the retirement transition in men and women: associations with emotion regulation, adjustment difficulties and work centrality
Source: BMC Geriatr. 2024 Jul 31;24:643. doi: 10.1186/s12877-024-05228-2 (PMC11292945; doi:10.1186/s12877-024-05228-2)
Supplement: Supplementary file 1 — Supplementary Material 1. Table S1. Descriptive statistics of differences in baseline (wave 1) characteristics between the HEARTS cohort and the study sample. Table S2. Interaction effects of gender differences in associations between depressive symptoms and emotion regulation, adjustment difficulties, and work centrality. Table S3. Gender-stratified associations between depressive symptoms and emotion regulation, adjustment difficulties, and work centrality. [file 12877_2024_5228_MOESM1_ESM.docx]

**Supplementary Material**

Depressive symptoms across the retirement transition in men and women: Associations with emotion regulation, adjustment difficulties and work centrality

**Table S1**. Descriptive statistics of differences in baseline (wave 1) characteristics between the study sample and individuals excluded from analyses due to gradual, reversed, indirect, or incomplete transition pathways.

|  | Study sample | Participants excluded from analyses^a^ | Group difference |
| --- | --- | --- | --- |
|  | M (SD) / % | M (SD) / % | *t/*$X^{2}$ |
| *n* | 527 | 726 |  |
| Gender (% women) | 56.4% | 57.6% | 0.14 |
| Age | 62.7 (1.6) | 62.9 (1.6) | 2.7** |
| Education years | 13.6 (3.4) | 13.3 (3.2) | -1.4 |
| Partner (% yes) | 81.3% | 85.3% | 3.18 |
| Partner working (% yes) | 55.6% | 58.8% | 1.10 |
| Self-rated functional health | 1.5 (0.6) | 1.5 (0.6) | 0.38 |
| CES-D score | 4.7 (4.5) | 4.6 (4.1) | -0.5 |

Note: ^a^Individuals who met the inclusion criteria (working and not retired at baseline and retiring during the study period) but were excluded because of gradual (*n* = 322), reversed (*n* = 134), indirect (from disability or unemployment, *n* = 52), or incomplete (drop-out during study period, *n* = 218) transition pathways. **p<0.01.

**Table S2**. Interaction effects of gender differences in associations between depressive symptoms and emotion regulation, adjustment difficulties, and work centrality.

|  | CES-D score | | | | |
| --- | --- | --- | --- | --- | --- |
|  | *b* (*SE*) | *b* (*SE*) | *b* (*SE*) | *b* (*SE*) | *b* (*SE*) |
| Time | -0.29*** (0.06) | -0.29*** (0.06) | -0.27*** (0.06) | -0.27** (0.06) | -0.27*** (0.06) |
| Time^2^ | 0.16*** (0.04) | 0.16*** (0.04) | 0.15*** (0.04) | 0.15*** (0.04) | 0.15***(0.04) |
| Time^3^ | 0.04*** (0.01) | 0.04*** (0.01) | 0.04*** (0.01) | 0.04*** (0.01) | 0.04*** (0.01) |
| Education | 0.08 (0.05) | 0.08 (0.05) | 0.05 (0.05) | 0.06 (0.05) | 0.05 (0.05) |
| Age | 0.02 (0.10) | 0.03 (0.10) | -0.02 (0.09) | -0.03 (0.09) | 0.03 (0,09) |
| Partner | -1.63*** (0.42) | -1.62*** (0.42) | -1.49*** (0.39) | -1.48*** (0.40) | -1.48*** (0.39) |
| Partner working | 0.53 (0.39) | 0.53 (0.39) | 0.54 (0.35) | 0.52 (0.36) | 0.53 (0.35) |
| Supression | 0.50* (0.22) | 0.41** (0.15) | 0.26 (0.14) | 0.25 (0.14) | 0.27 (0.14) |
| Reappraisal | -0.46** (0.15) | -0.46 (0.26) | -0.32* (0.15) | -0.32* (0.15) | -0.32* (0.15) |
| Adjustment difficulties |  |  | 1.59*** (0.40) | 2.13*** (0.28) | 2.12*** (0.28) |
| Importance of work for self-esteem |  |  | 0.50*** (0.13) | 0.36 (0.19) | 0.51*** (0.13) |
| Meaning of work |  |  | -0.99*** (0.19) | -0.99*** (0.19) | -1.40*** (0.27) |
| Gender (male = 0, female = 1) | 0.80* (0.34) | 0.79* (0.34) | 1.17*** (0.31) | 1.15*** (0.31) | 1.19*** (0.31) |
| *Interaction effects* |  |  |  |  |  |
| Gender x Supression | -0.17 (0.29) |  |  |  |  |
| Gender x Reappraisal |  | 0.004 (0.32) |  |  |  |
| Gender x Adjustment difficulties |  |  | 1.03 (0.55) |  |  |
| Gender x Importance of work for self-esteem |  |  |  | 0.24 (0.25) |  |
| Gender x Meaning of work |  |  |  |  | 0.72 (0.36)* |
| Constant | 4.15*** (0.42) | 4.17*** (0.42) | 3.88*** (0.40) | 3.85*** (0.40) | 3.80*** (0.40) |
| Observations | 2183 | 2183 | 2073 | 2073 | 2073 |
| Log likelihood | -5,659.41 | 5,659.47 | -5,317.77 | -5,319.83 | 5,317.93 |
| Akaike information criterion | 11,346.82 | 11,346.95 | 10,669.55 | 10,673.67 | 10,669.86 |
| Bayesian information criteron | 11,426.46 | 11,426.59 | 10,765.37 | 10,769.49 | 10,765.68 |

Note. *p<0.05; **p<0.01; ***p<0.001.

**Table S3.** Gender-stratified associations between depressive symptoms and emotion regulation, adjustment difficulties, and work centrality.

|  | Men | | | | Women | | | |
| --- | --- | --- | --- | --- | --- | --- | --- | --- |
|  | Model 1 | Model 2 | Model 3 | Model 4 | Model 1 | Model 2 | Model 3 | Model 4 |
|  | *b* (*SE*) | *b* (*SE*) | *b* (*SE*) | *b* (*SE*) | *b* (*SE*) | *b* (*SE*) | *b* (*SE*) | *b* (*SE*) |
| Time | -0.22**  (0.07) | - 0.24**  (0.08) | -0.22**  (0.08) | -0.19*  (0.07) | -0.32***  (0.08) | -0.33***  (0.08) | -0.31***  (0.08) | -30***  (0.09) |
| Time^2^ | 0.14**  (0.05) | 0.12*  (0.05) | 0.11*  (0.05) | 0.10*  (0.05) | 0.17**  (0.05) | 0.18***  (0.05) | 0.19***  (0.05) | 0.19***  (0.05) |
| Time^3^ | 0.03**  (0.01) | 0.03**  (0.01) | 0.03**  (0.01) | 0.03*  (0.01) | 0.04**  (0.01) | 0.04***  (0.01) | 0.04***  (0.01) | 0.04**  (0.01) |
| Education | 0.07  (0.07) | 0.08  (0.07) | 0.10  (0.06) | 0.04  (0.05) | -0.03  (0.07) | 0.05  (0.08) | -0.04  (0.07) | -0.05  (0.06) |
| Age | 0.02  (0.14) | -0.09  (0.15) | -0.11  (0.14) | -0.22*  (0.11) | 0.10  (0,13) | 0.11  (0.13) | 0.04  (0.12) | 0.11  (0.11) |
| Partner | -1.64*  (0.65) | -1.70**  (0.66) | -1.29*  (0.61) | -0.34  (0.52) | -1.79***  (0.54) | -1.46**  (0.56) | -1.51**  (0.53) | -0.94  (0.49) |
| Partner working | 0.17  (0.51) | 0.30  (0.53) | 0.41  (0.48) | 0.31  (0.39) | 0.75  (0.57) | 0.84  (0.61) | 0.60  (0.56) | 0.36  (0.49) |
| Suppression |  | 0.52*  (0.22) | 0.36  (0.20) | -0.005  (0.17) |  | 0.33  (0.20) | 0.17  (0.19) | -0.07  (0.17) |
| Reappraisal |  | -0.48  (0.26) | -0.38  (0.24) | -0.20  (0.19) |  | -0.43*  (0.19) | -0.26  (0.19) | -0.10  (0.17) |
| Adjustment difficulties |  |  | 1.66***  (0.40) | 1.06**  (0.33) |  |  | 2.65***  (0.39) | 1.66***  (0.37) |
| Importance of work for self-esteem |  |  | 0.44*  (0.19) | 0.30  (0.16) |  |  | 0.52**  (0.17) | 0.37*  (0.15) |
| Meaning of work |  |  | -1.34***  (0.28) | -0.95***  (0.23) |  |  | -0.70**  (0.25) | -0.56*  (0.23) |
| Social support |  |  |  | -1.00***  (0.15) |  |  |  | -0.90***  (0.16) |
| Self-rated functional health |  |  |  | 1.08***  (0.31) |  |  |  | 1.86***  (0.32) |
|  |  |  |  |  |  |  |  |  |
| Constant | 4.66***  (0.55) | 4.67***  (0.55) | 4.32***  (0.52) | 3.60***  (0.45) | 4.86***  (0.50) | 4.51***  (0.51) | 4.56***  (0.49) | 4.13***  (0.46) |
|  |  |  |  |  |  |  |  |  |
| Observations | 1,015 | 962 | 915 | 891 | 1,296 | 1,221 | 1,158 | 1,102 |
| Log likelihood | -2,529.74 | -2,399.38 | -2,248.17 | -2,125.62 | -3,440.92 | -3,247.78 | -3,053.14 | -2,853.65 |
| Akaike information criterion | 5,079.48 | 4,822.77 | 4,526.33 | 4,285.24 | 6,901.84 | 6,519.57 | 6,136.27 | 5,741.30 |
| Bayesian information criteron | 5,128.71 | 4,881.20 | 4,598.62 | 4,366.71 | 6,953.51 | 6,580.86 | 6,212.09 | 5,826.39 |

Note. *p<0.05; **p<0.01; ***p<0.001
